# Supplementary material for: Adolescent Alcohol Drinking Renders Adult Drinking BLA-Dependent: BLA Hyper-Activity as Contributor to Comorbid Alcohol Use Disorder and Anxiety Disorders
Source: Brain Sci. 2017 Nov 14;7(11):151. doi: 10.3390/brainsci7110151 (PMC5704158; doi:10.3390/brainsci7110151)
Supplement: Supplementary file 1 [file brainsci-07-00151-s001.pdf]

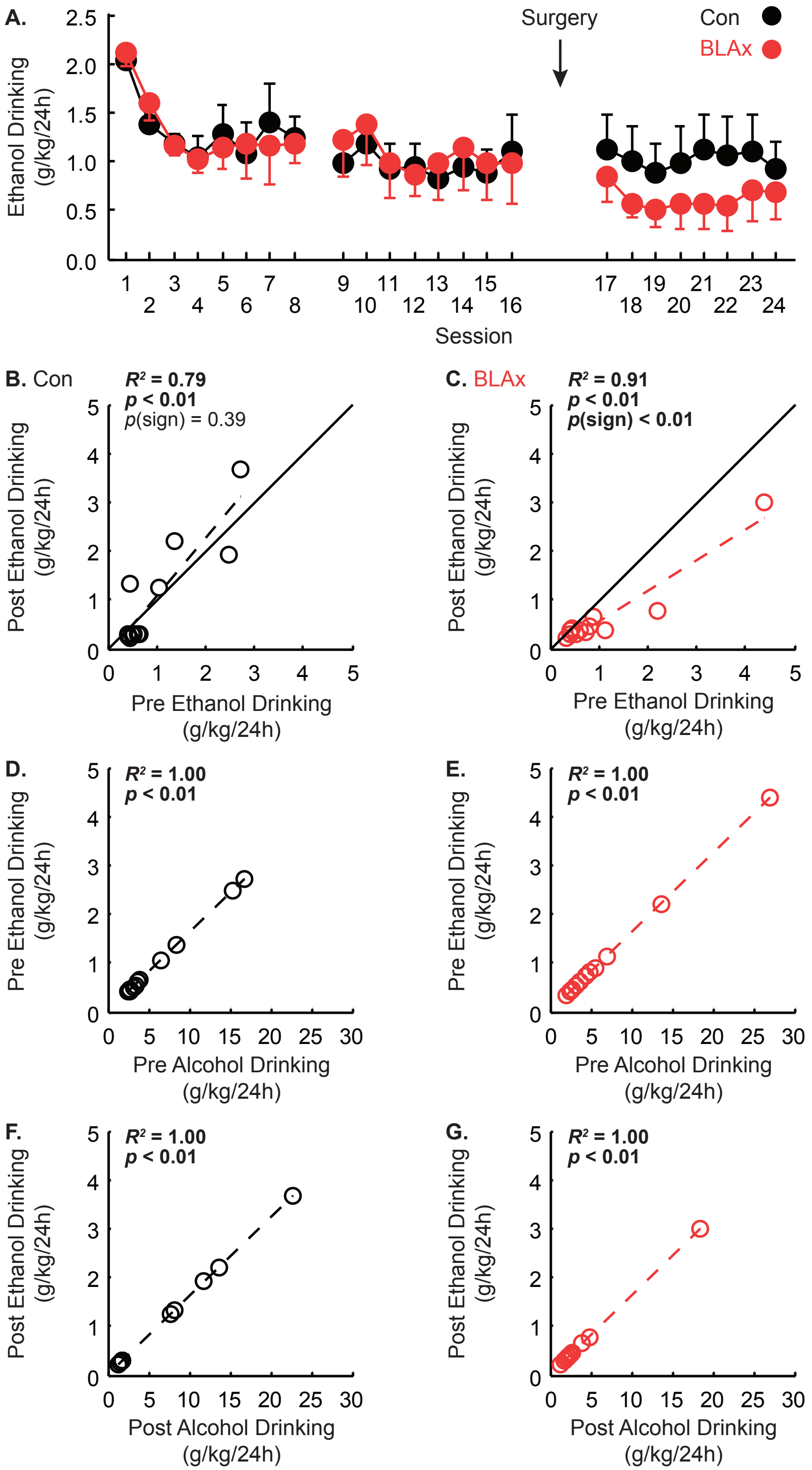

Figure S1. (**A**) Plotting ethanol drinking exactly as for alcohol drinking (Figure 2A) reveals an identical statistical pattern on a reduced y-axis scale. In support, ANOVA for ethanol drinking with lesion (Control vs. BLAx) and session (9-24) as factors, found a significant lesion x session interaction identical to that obtained for alcohol drinking ($F_{15,330}$ = 2.05, p = 0.01). The relationship between pre-surgery and post-surgery ethanol drinking across all individuals for was identical to that obtained for alcohol drinking for (**B**) Controls (compare to Figure 3A) and (**C**) BLAx (compare to Figure 3C). Pre-surgery alcohol drinking (x-axis) and pre-surgery ethanol drinking (y-axis) were perfectly correlated for (**D**) Controls and (**E**) BLAx. Perfect correlations were also obtained for post-surgery drinking in (**F**) Controls and (**G**) BLAx.
